# Flame retardant properties and mechanism of PLA/P-PPD -Ph /ECE conjugated flame retardant composites

**DOI:** 10.3389/fchem.2023.1096526

**Published:** 2023-03-16

**Authors:** Yanyan Tan, Daohai Zhang, Yu Xue, Xiao Zhan, Fang Tan, Shuhao Qin

**Affiliations:** ^1^ School of Chemical Engineering of Guizhou Minzu University, Guiyang, China; ^2^ National Engineering Research Center for Compounding and Modification of Polymer Materials, Guiyang, China

**Keywords:** ECE, P-PPD-Ph, thermal performance, flame retardancy, mechanical property

## Abstract

In this article, 4, 4'-{1'',4''-phenylene-bis[amido-(10'' ''-oxo-10'''-hydro-9'''-oxa-10'''λ5-phosphafi-10'''-yl)-methyl]}-diphenol (P-PPD-Ph) was synthesized by a two-step synthesis, followed by the addition of various levels of epoxy chain extender (ECE) with 5 wt% of P-PPD-Ph The PLA/P-PPD-Ph/ECE conjugated flame retardant composites were produced by co-extrusion into poly(lactic acid) (PLA). The chemical structure of P-PPD-Ph was characterized by FTIR, ^1^H NMR and ^31^P NMR tests, demonstrating the successful synthesis of the phosphorus heterophilic flame retardant P-PPD-Ph. The structural, thermal, flame retardant and mechanical properties of the PLA/P-PPD-Ph/ECE conjugated flame retardant composites were characterised using FTIR, thermogravimetric analysis (TG), vertical combustion testing (UL-94), limiting oxygen index (LOI), cone calorimetry, scanning electron microscopy (SEM), elemental energy spectroscopy (EDS) and mechanical properties testing. The structural, thermal, flame retardant and mechanical properties of PLA/P-PPD-Ph/ECE conjugated flame retardant composites were characterised. The results showed that with the increase of ECE content, the residual carbon rate of the composites increased from 1.6% to 3.3%, and the LOI value increased from 29.8% to 32.6%. The cross-linking reaction between P-PPD-Ph and PLA and the increase of reaction sites led to the generation of more phosphorus-containing radicals on the PLA molecular chain, which strengthened the cohesive phase flame retardant effect of PLA flame retardant composites, and The bending strength, tensile strength and impact strength were all improved.

## 1 Introduction

Polylactic acid (PLA), as a novel biodegradable material, has attracted a great deal of attention due to its excellent thermal stability, biocompatibility and degradability and is widely used in food packaging and biopharmaceutical products (Arrieta et al., 2013; Khankrua et al., 2014; Cayla et al., 2016; Qiu et al., 2017). However, PLA’s inherent degradability and flammability may hinder PLA’s use in the manufacturing and processing process, therefore, flame retardant modification of PLA materials is of particular importance (Xi et al., 2016). Due to the small number of PLA molecular chain branches, the molecular structure with terminal hydroxyl or terminal carboxyl groups, there is thermal degradation, oxidation and hydrolysis during processing, which can easily cause molecular chain breakage and further reduce the melt viscosity, so PLA can be processed by adding the appropriate amount of chain extender and flame retardant to reduce the hydrolysis and oxidation of PLA molecular chain, improve the processing stability, and achieve improved mechanical properties, thermal properties and Crystallization properties (Salmeia and Gaan, 2015). Sun et al. (2022); Basak and Guralp (2017) modified PLA with ADR-4368 and showed that the addition of an epoxy chain extender improved the mechanical and processing properties of PLA as well as the thermal stability of PLA.

Along with improved processing stability, it is important to improve flame resistance. 9,10-dihydro-9-oxa-10- phosphaphenanthrene-10-oxide (DOPO)-derived flame retardants have been widely used in polyester, polyamide and epoxy resins and other polymer materials due to the advantages of low addition, good thermal stability and low impact on the overall performance of the polymer (Yang et al., 2015; Du et al., 2019; Liu et al., 2022). P-PPD-Ph is a new type of DOPO-derived flame retardant with a high number of aromatic groups in its structure, which makes P-PPD-Ph have better thermal stability due to its conjugation and “cage effect” in the process of thermal degradation. The functional modification of PLA by adding flame retardants can improve its flame retardant properties, making PLA biodegradable material can be used in electronic and electrical appliances, automotive, construction, textile and other fireproof products (Chokwe et al., 2020; Sun et al., 2022; Zhang et al., 2022).

In this study, we prepared PLA/P-PPD-Ph/ECE conjugated flame retardant composites by adding different additions of epoxy chain extender ECE with 5 wt% of the prepared P-PPD-Ph to the PLA matrix. It was found that different additions of ECE could improve the thermal, flame retardant and mechanical properties of PLA/P-PPD-Ph/ECE conjugated flame retardant composites. This is because ECE acts as a “bridge” in the reaction between PLA and phosphorus heterophilic flame retardants, providing more phosphorus-containing flame retardant reactive groups for the reaction between P-PPD-Ph and PLA matrix, which is a novel feature of the article.

## 2 Materials and methods

### 2.1 Materials

DOPO (analytical purity), purchased from Beijing Huawei Rike Chemical Co., Ltd.; ethanol (analytical purity), purchased from Chongqing Jiangchuan Chemical Co., Ltd.; p-hydroxybenzaldehyde (analytical purity), p-phenylenediamine purchased from (analytical purity), Tianjin Comio Chemical Reagent Co.; Polylactic acid (model 4032D), purchased from Natureworks, United States; P-PPD-Ph (laboratory preparation); epoxy chain extender (ECE) (model ADR-5481), purchased from Sinopharm Chemical Reagent Co.

### 2.2 Synthesis of P-PPD-Ph compounds

**FIGURE 1:**
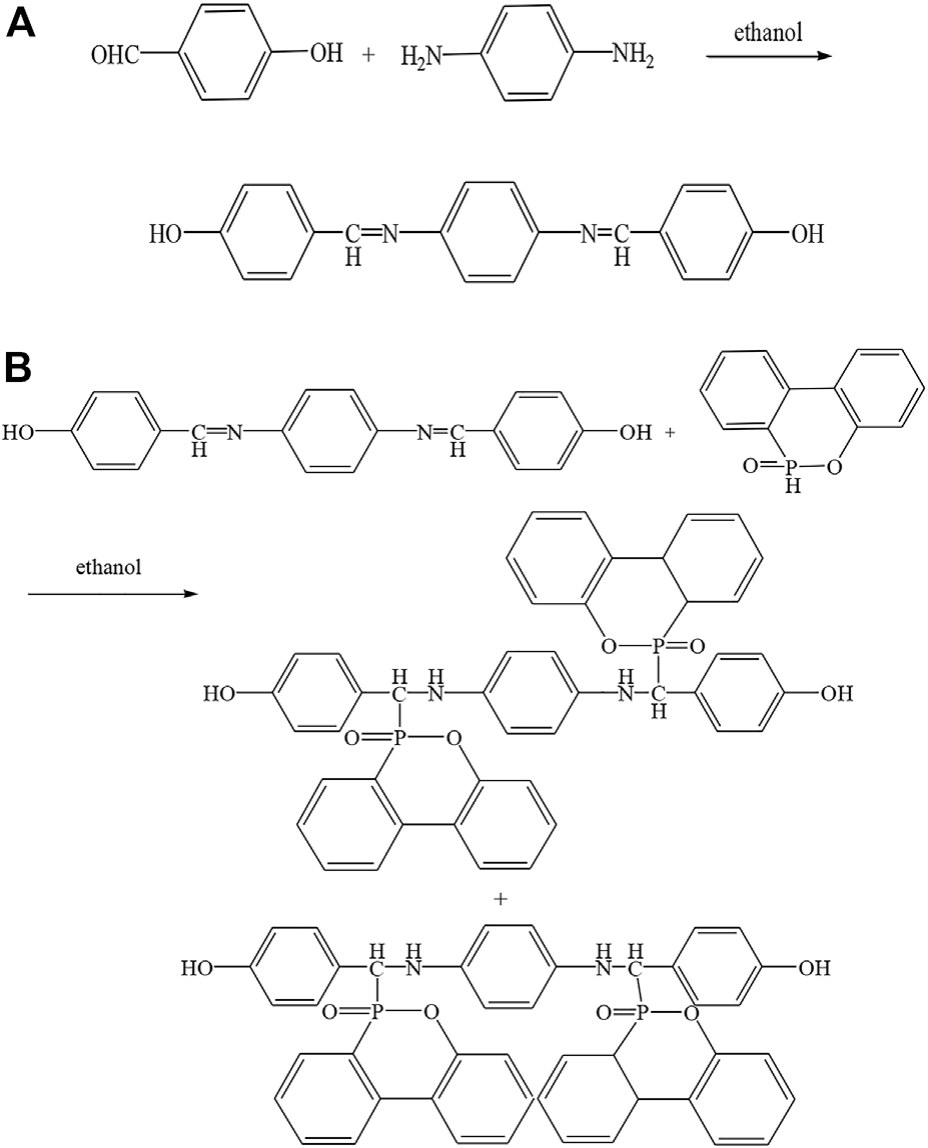
Synthetic chemical equation of **(A)** the imine compounds and **(B)** P-PPD-Ph.

The synthesis of P-PPD-Ph was divided into two steps and its synthesis method was referenced from the literature. The first step was the synthesis of the intermediate imine structure compound and the synthesis scheme of the imine compound is shown in Figure 1A. The synthesis was carried out by adding p-phenylenediamine (0.10 mol 10.814 g), p-hydroxybenzaldehyde (0.20 mol, 24.424 g) and 200 mL of anhydrous ethanol to a 500 mL three-necked glass flask equipped with a reflux device and a mechanical stirring device. The mixture was warmed to 50°C under nitrogen and the reaction was stirred for 2 h. As the reaction proceeded, the product precipitated as a pale yellow precipitate. The mixture was cooled to room temperature, the pale yellow precipitate was filtered and washed several times with ethanol, then dried in a vacuum oven at 80°C for 8 h to give 31.01 g of pale yellow crystals (88% yield). The second step was the synthesis of P-PPD-Ph. The synthesis scheme of P-PPD-Ph is shown in Figure 1B. The synthesis procedure was as follows: DOPO (0.20 mol, 43.237 g), imine compound (0.10 mol, 31.6 g) and 300 mL of anhydrous ethanol were added to a 500 mL three-necked glass flask equipped with a condensing device and a mechanical stirrer. The reaction mixture was stirred at 50°C for 10 h and then the mixture was cooled to room temperature. The yellow precipitate was filtered and washed several times with ethanol and then dried in a vacuum oven at 80°C for 8 h. After drying, 31.61 g of light yellow crystals were obtained (88% yield).

### 2.3 Preparation of flame retardant PLA materials

The PLA, ECE and P-PPD-Ph flame retardants (fixed content of 5wt%) were vacuum dried at 80°C for 4 h. Five mixed systems were prepared by mixing PLA, P-PPD-Ph and 0 wt‰, 2 wt‰, 4 wt‰, 6 wt‰, and 8 wt‰ ECE respectively. The twin-screw extruder (CTE-20, Coperion Machinery Co., Ltd., China) is used to extrude at the temperature range of 180°C–200°C and the screw speed of 300 rpm. The extruded granules (SE-130; DongHua Machinery Co., Ltd., China) are molded into samples. The injection molding machine is used to inject at 180°C–200°C to prepare standard samples, which are respectively marked as PLA/P-PPD-Ph/ECE—0 wt‰, PLA/P-PPD-Ph/ECE—2 wt‰, PLA/P-PPD-Ph/ECE—4 wt‰, PLA/P-PPD-Ph/ECE—6 wt‰, PLA/P-PPD-Ph/ECE—8 wt‰.

### 2.4 Characterization methods

A Fourier transform infrared spectrometer (Nicolet 1N10MX) was used to characterise the structure of the prepared P-PPD-Ph phosphorus heterophilic flame retardant and PLA composites, and a nuclear magnetic resonance instrument (model Bruker 400, Bruker, Switzerland) was used for further characterisation of the structure of the prepared P-PPD-Ph. Specifically, 1 mg of the sample was dissolved in dimethyl sulfoxide (DMSO-d6) and tested at a test range of −200 to 200 ppm to obtain ^1^H NMR and ^31^P NMR spectra.

The thermal properties of the prepared P-PPD-Ph and four kinds of PLA composites were tested with a thermogravimetric analyzer (model Q50, American TA Company) to analyze the thermal stability and thermal degradation behavior of the materials.

SH5300 vertical combustion test (UL-94) (Guangzhou Xinhe Electronic Equipment Co., Ltd.), JF-3 limiting oxygen index test (LOI) (Nanjing Jiangning District Analytical Instrument Co., Ltd.) and FTT cone calorimeter (American Fire Testing Technology Co., Ltd.) were used for flame retardancy test. UL-94 test conforms to GB/T 2408-1996, and the sample size is 125*13*3 mm^3^. LOI test conforms to GB/T 2406-1993 and the size of the spline is 80*10*4 mm^3^. The cone calorimeter test conforms to ISO5660-1 and the size of the spline is 100*100*6 mm^3^.

A Scanning electron microscope (model FEI Quanta 250, FEI Company, United States) and an element energy spectrum analyzer were used to analyze the morphology and element content of the carbon layer surface of four PLA composites after combustion.

The tensile property, bending property and strength of four kinds of PLA composites prepared were tested with a microcomputer controlled electronic universal testing machine according to GB/T 1040.1-2006, GB/T 9341-2008 and GB/T 1843-2008.

## 3 Results and discussion

### 3.1 Infrared spectroscopy (FTIR) analysis of P-PPD-Ph

**FIGURE 2:**
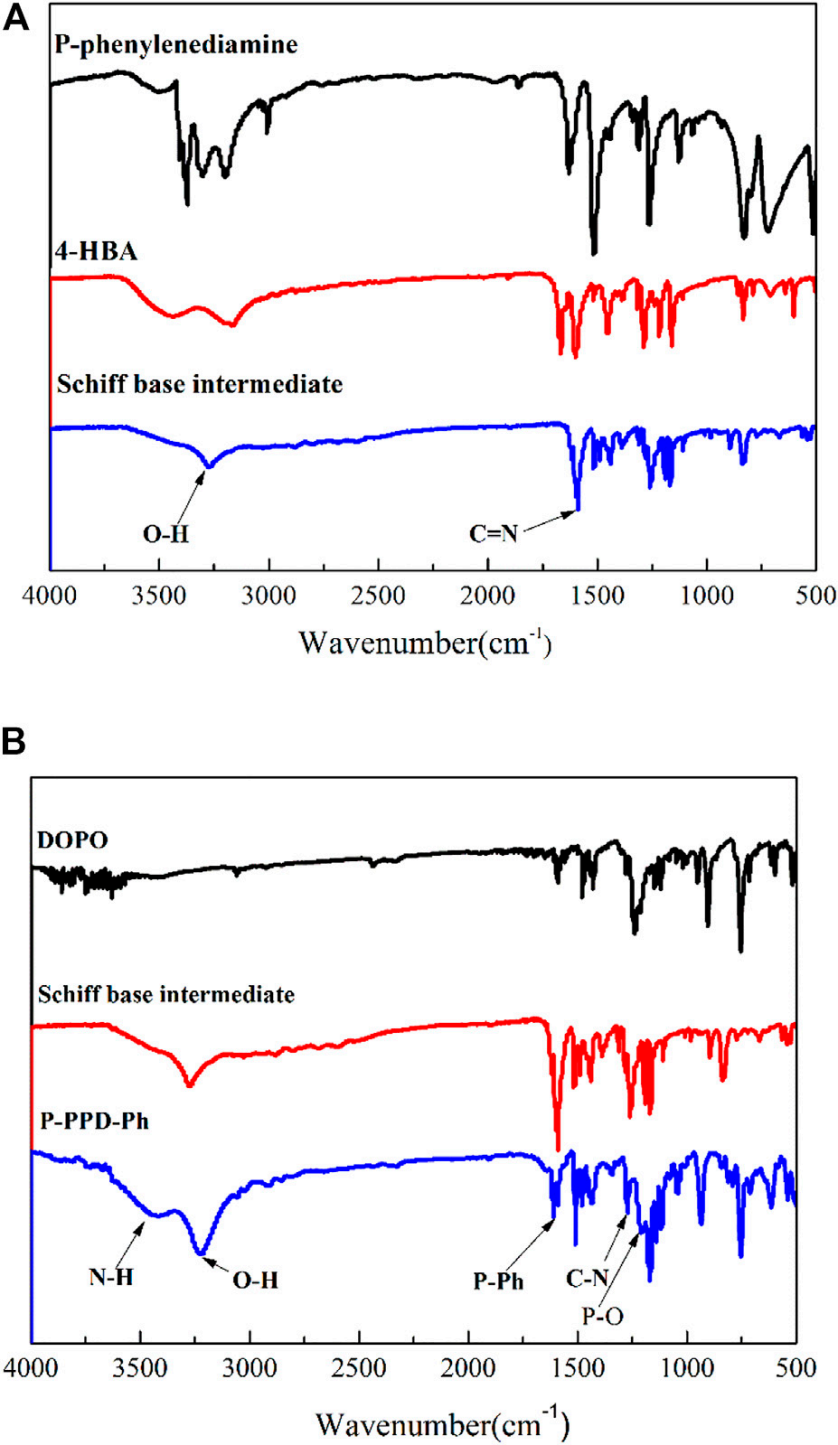
FTIR spectrum of **(A)** imine compounds and **(B)** P-PPD-Ph.

Figure 2A shows the FTIR spectra of p-hydroxybenzaldehyde, p-phenylenediamine and the synthesised Schiff base intermediate compounds. In the IR spectrum of the Schiff base intermediate, the characteristic peaks are at 1605 cm^-1^ for C=N and 3274 cm^-1^ for-OH. Figure 2B then shows the FTIR spectra of P-PPD-Ph and the DOPO and imine compounds. In the IR spectrum of P-PPD-Ph, the characteristic peaks are O-H at 3230 cm^-1^, P-Ph at 1599 cm^-1^, C-N at 1277 cm^-1^, P (=O)-O-C at 1213 cm^-1^ and P-O-Ph at 929 cm^-1^. And the disappearance of the P-H bond at position 2434 cm^-1^ can be observed in the IR spectrum of P-PPD-Ph, indicating that DOPO has chemically reacted with the Schiff base intermediate.

### 3.2 Nuclear magnetic resonance spectroscopy (NMR) analysis of P-PPD-Ph

**FIGURE 3:**
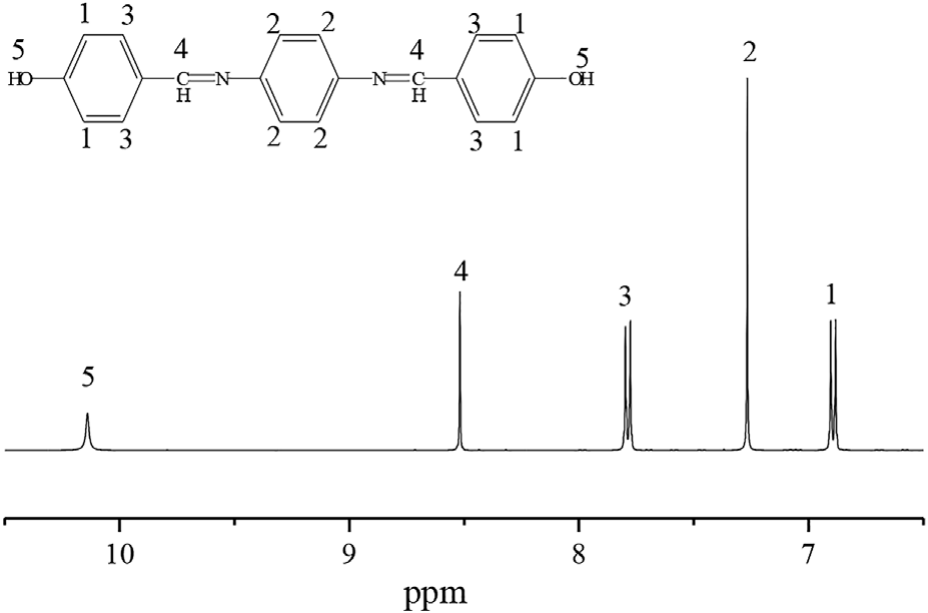
^1^H NMR spectrum of the intermediate imine structure.

The ^1^ H NMR spectrum of the Schiff base intermediate is shown in Figure 3. The attribution of the individual peaks in the ^1^ H-NMR spectrum of the Schiff base intermediate corresponds to the following: *δ* = 6.88 (H1), 6.90 (H1), 7.26 (H2), 7.77 (H3), 7.79 (H3), 8.51 (H4), and 10.13 (H5), with peak chemical shifts and integral areas consistent with the product structure.

**FIGURE 4:**
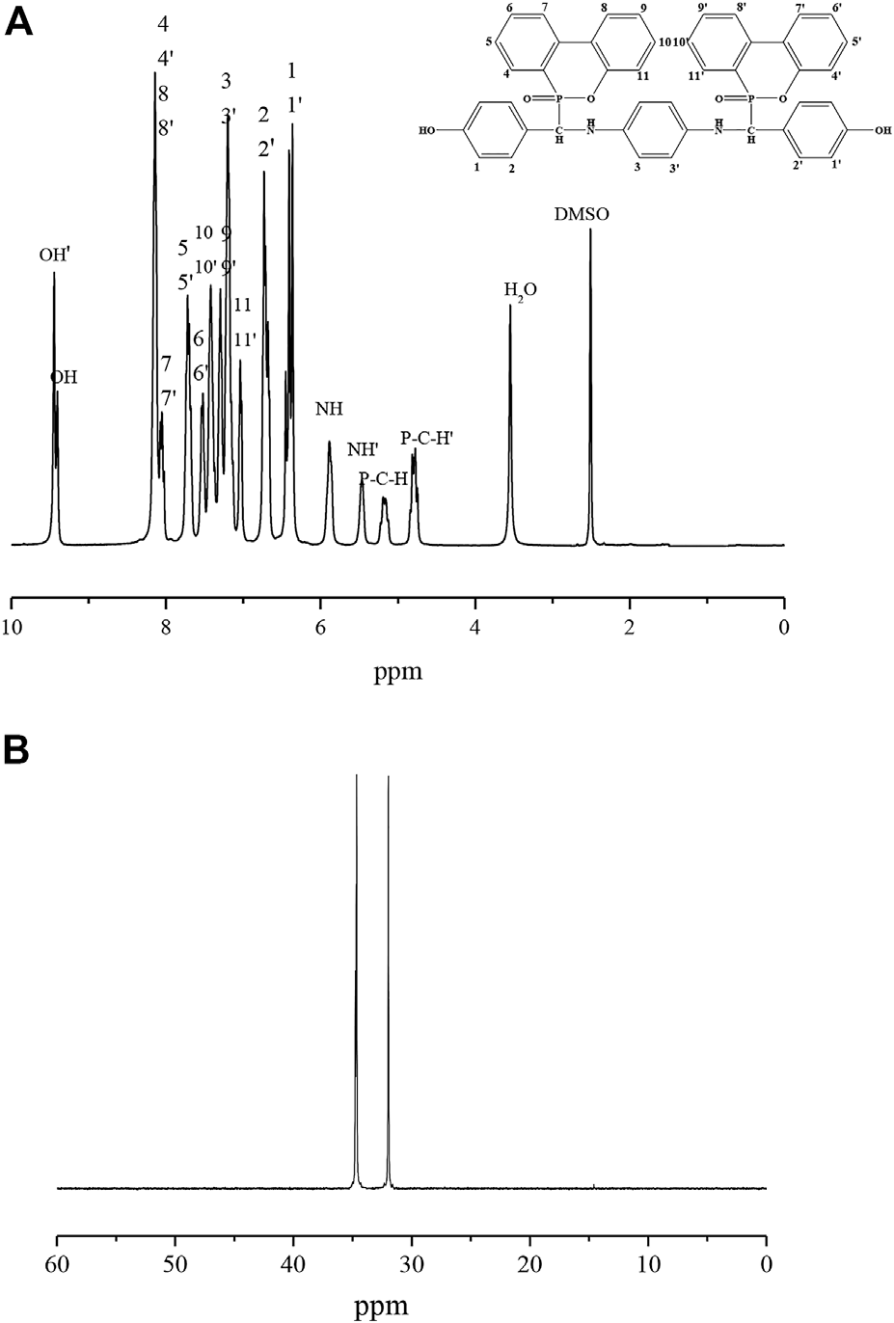
^1^H NMR spectrum **(A)** and ^31^ P NMR spectrum of P-PPD-Ph **(B)**.

The ^1^ H NMR and ^31^ P NMR spectra of P-PPD-Ph are shown in Figure 4. Figure 4A shows the ^1^ H NMR spectrum of P-PPD-Ph. The individual peaks in the ^1^ H NMR spectrum of P-PPD-Ph are attributed as follows: 4.74–4.84 (PC-H′), 5.12–5.22 (PCH), 5.46 (NH’), 5.88 (NH), 6.26–6.45 (1,1′), 6.67–6.73 (2,2′) 7.04 (11,11′), 7.20 (3,3′), 7.29 (9,9′), 7.42 (10,10′), 7.53 (6,6′), 7.68–7.72 (5,5′), 8.02–8.07 (7,7′), 8.10–8.14 (4,4′8,8′), 9.40 (OH), and 9.44 (OH’), and The integrated area ratios of the peaks are in general agreement with the ratio of the number of hydrogen atoms in the P-PPD-Ph structure. Figure 4B shows the ^31^ P NMR spectrum of P-PPD-Ph, as shown in the figure, the phosphorus atom resonance peaks at 31.64 and 34.66 ppm, respectively, due to the presence of isomers in the structure of P-PPD-Ph (as shown in Figure 1), which was also reported in other similar literature. And the ^1^ H NMR spectrum of P-PPD-Ph also further demonstrated the presence of isomers in the structure of P-PPD-Ph.

### 3.3 Structural analysis of PLA/P-PPD-Ph/ECE conjugated flame retardant composites

**FIGURE 5:**
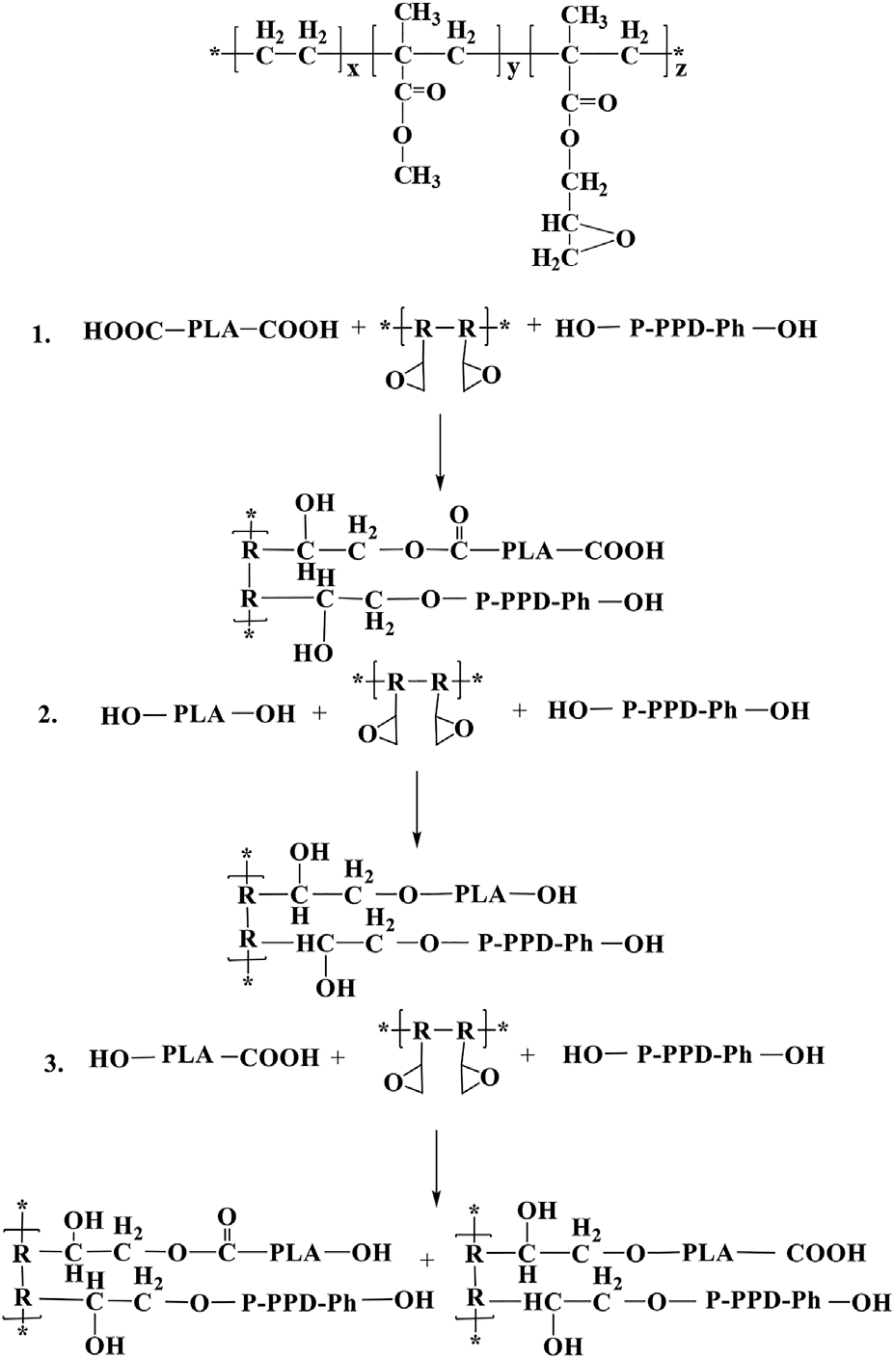
Structural formula of ECE and major chemical reactions that may occur in PLA/ECE/P-PPD-Ph Conjugated flame retardant composites.

**FIGURE 6:**
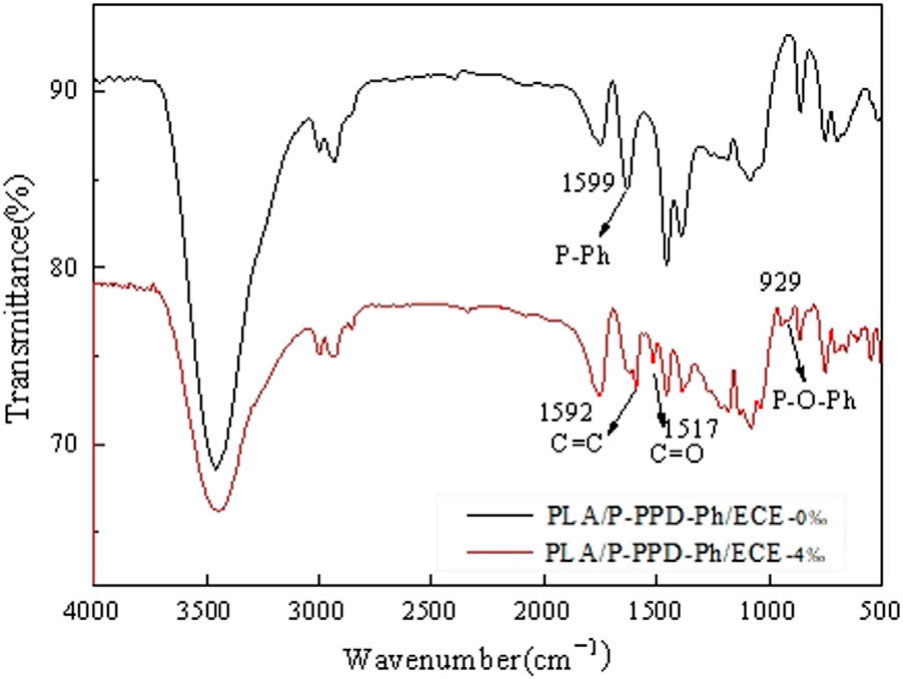
FTIR spectra of PLA/P-PPD-Ph/ECE conjugated flame retardant composites.

The PLA matrix material, ECE and the flame retardant P-PPD-Ph all carry reactive functionalities. The reactive end hydroxyl/carboxyl groups in PLA, the active hydroxyl groups in the P-PPD-Ph structure and the epoxy groups in the ECE structure may undergo complex chemical reactions during the mixing and processing into standard samples. Figure 5 shows the structural formula of ECE and the main chemical reactions that can occur in the three components of the PLA/P-PPD-Ph/ECE conjugated flame retardant composite (Buczko et al., 2014; Ming et al., 2022). The FTIR of the flame retardant material is shown in Figure 6. The plot of PLA/P-PPD-Ph/ECE-0% corresponds to the absorption peak of P-Ph at 1599 cm^-1^. Compared to the FTIR absorption peaks of PLA/P-PPD-Ph/ECE-0‰, a new IR absorption peak appears on the plot of PLA/P-PPD-Ph/ECE-4‰, that is, 1592, 1517, and 929 cm^-1^ positions corresponding to the C=C, C=O, and P-O-Ph absorption peaks, respectively, indicating that the flame retardants P-PPD-Ph, PLA, and ECE underwent a chemical reaction during the mixing process and therefore new chemical bonds were created.

### 3.4 Analysis of the thermal properties of PLA/P-PPD-Ph/ECE conjugated flame retardant composites

**FIGURE 7:**
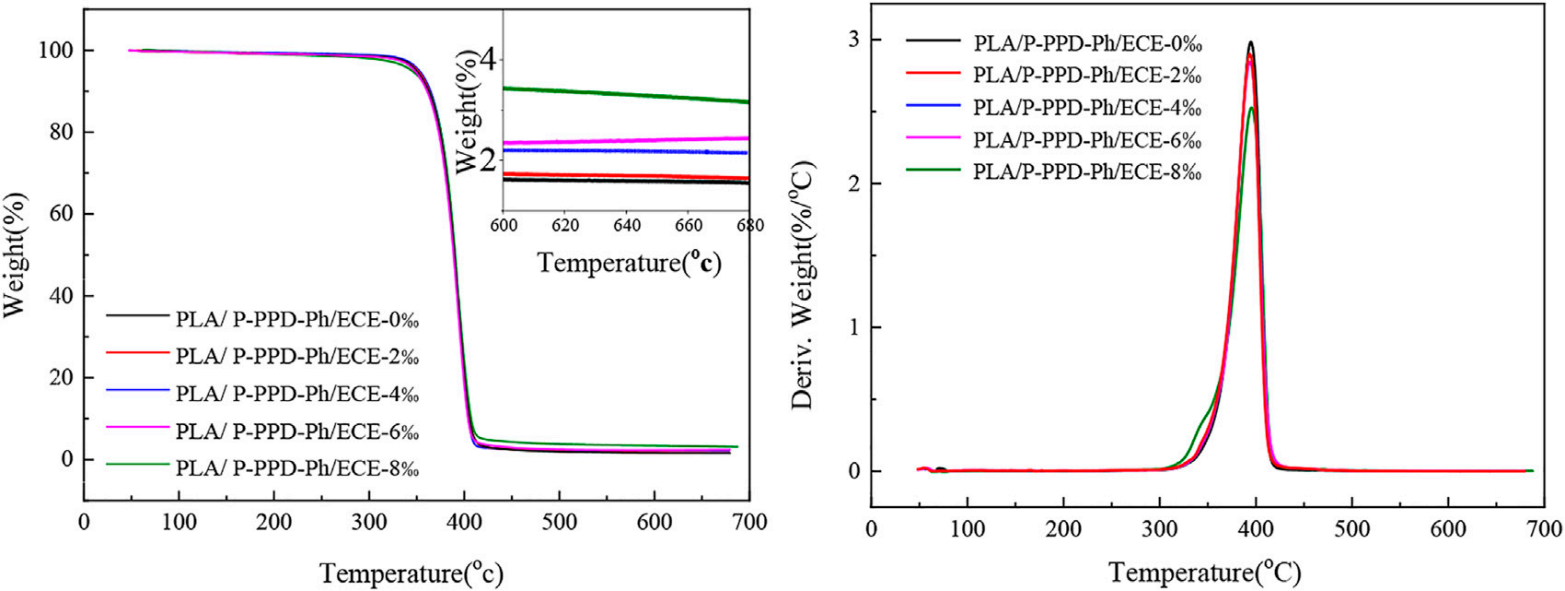
TGA and DTG curves of PLA/P-PPD-Ph/ECE conjugated flame retardant composites.

The TGA and DTG curves of PLA/P-PPD-Ph/ECE conjugated flame retardant composites under nitrogen are shown in Figure 7 and the corresponding data are listed in Table 1. The initial decomposition temperature of PLA flame retardant composites increased with the addition of ECE. The initial decomposition temperature of PLA/P-PPD-Ph/ECE flame retardant composites was 344.6°C without the addition of ECE, and increased to 349.0°C with the addition of 8 wt‰ of ECE. Moreover, the addition of ECE relatively reduces the maximum thermal decomposition rate of PLA flame retardant composites, and the maximum decomposition rate of flame retardant composites decreases from 2.9%/°C to 2.8%/°C with the addition of ECE at 8 wt‰. The maximum thermal decomposition temperature and the amount of residual carbon of the PLA/P-PPD-Ph/ECE conjugated flame retardant composites increased as the addition of ECE increased. When the addition of ECE was 8 wt‰, the maximum thermal decomposition temperature of PLA flame retardant composites increased from 394.0°C to 395.9°C and the residual carbon rate around 700°C increased from 1.6% to 3.3%. The addition of ECE improved the thermal stability of PLA/P-PPD-Ph/ECE conjugated flame retardant composites. This is mainly due to the inverse chemical reaction and cross-linking of ECE with the PLA matrix and the flame retardant P-PPD-Ph during processing, which hinders the thermal degradation of the composite (Sun and Yao, 2011).

**TABLE 1:** TGA and DTG data for PLA/P-PPD-Ph/ECE conjugated flame retardant composites.

Sample	T_-5%_ (°C)	T_max_ (°C)	The value at T_max_ (%/°C)	Residues
PLA/P-PPD-Ph/ECE-0‰	344.6	394.0	2.9	1.6
PLA/P-PPD-Ph/ECE-2‰	345.2	394.6	2.8	1.7
PLA/P-PPD-Ph/ECE-4‰	345.7	394.8	2.8	2.2
PLA/P-PPD-Ph/ECE-6‰	347.4	395.1	2.8	2.3
PLA/P-PPD-Ph/ECE-8‰	349.0	395.9	2.8	3.3

### 3.5 Flame retardancy analysis of PLA/P-PPDPh/ECE conjugated flame retardant composites

In order to assess the effect of ECE on the flame retardancy of PLA/P-PPD-Ph/ECE conjugated flame retardant composites, the flame retardancy was tested using the UL-94 test and the LOI test. The UL-94 test ratings and LOI test results for PLA/P-PPD-Ph/ECE conjugated flame retardant composites are shown in Table 2. All composites can achieve a UL-94 V-0 rating. Ceren et al. (2022) studied the flame retardant effect of Joncryl epoxy chain extender (CE) on PLA flame retardant composites and the V0 value was only achieved when 1 wt% CE was used, although CE could contribute to the improvement of the fire resistance of PLA composites, the enhancement of the flame retardant properties of PLA composites by ECE was more obvious. In terms of oxygen index, the value of LOI increased from 29.8% to 32.6% after the addition of ECE. In addition, as the amount of ECE added increased, the time (t_1_/t_2_) that the sample burns after being ignited first increases and then tended to decrease., and the burning time of PLA/P-PPD-Ph/ECE-4‰ was relatively longer, which indicates that the addition of ECE in the right amount does significantly enhance the flame retardant properties of PLA/P-PPD-Ph/ECE conjugated flame retardant composites, while the effect of adding more ECE is not obvious. This is because when ECE is added at 4‰, the reaction sites between P-PPD-Ph and PLA not only increase, which improves the reaction rate between the two, but also increases the crosslinking of PLA molecular chains, thus making the PLA/P-PPD-Ph/ECE conjugated flame retardant composites have better flame retardant effect. The resulting PLA/P-PPD-Ph/ECE conjugated flame retardant composite can therefore be used as a promising durable bioplastic engineering product where fire risks exist.

**TABLE 2:** Test results of UL-94 and LOI.

Samples	LOI(%)	UL-94 (3.2 mm)			
		t _1_/t _2_s)	Dripping	Ignition	Rating
PLA/P-PPD-Ph/ECE-0‰	29.8	6.78/4.56	Yes	No	V-0
PLA/P-PPD-Ph/ECE-2‰	30.8	7.65/3.36	Yes	No	V-0
PLA/P-PPD-Ph/ECE-4‰	31.4	3.45/0.23	Yes	No	V-0
PLA/P-PPD-Ph/ECE-6‰	32.0	4.37/0.78	Yes	No	V-0
PLA/P-PPD-Ph/ECE-8‰	32.6	3.64/1.23	Yes	No	V-0

t_1_, average combustion times after the first application of the flame; t_2_, average combustion times after the second application of the flame.

**FIGURE 8:**
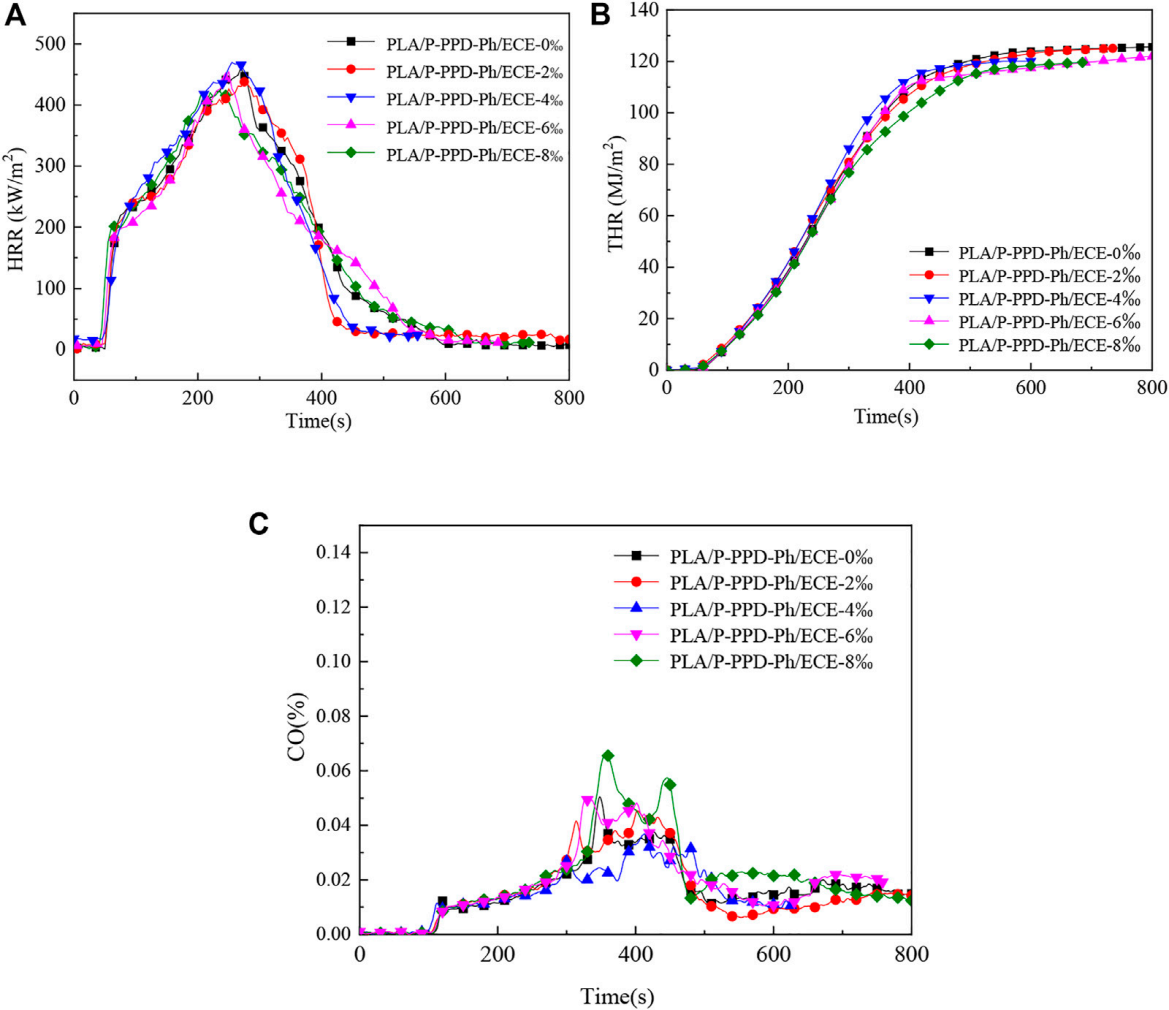
Cone calorimeter test curves for PLA/P-PPD-Ph/ECE conjugated flame retardant composites, **(A)** HRRcurve, **(B)** THR curve, **(C)** CO release curve.

Cone calorimetry tests were used to further analyse the flame retardant properties of ECE on PLA/P-PPD-Ph/ECE conjugated flame retardant composites. The test results are shown in Figure 8 and Table 3. The ignition time (TTI) of the PLA flame retardant composites did not change much with the addition of ECE and with the increase of the addition amount. As seen in Figure 8A, C and Table 3, ECE has a relatively large effect on heat release rate (HRR) and peak heat release rate (PHRR), with the value of PHRR decreasing from 452 kW/m^2^ to 419 kW/m^2^ when the addition of ECE is increased to 8 wt‰, a 7.3% decrease compared to the material without the addition of ECE. The addition of ECE also reduced the total heat release (THR) values of the PLA/P-PPD-Ph/ECE conjugated flame retardant composites, with little effect on the THR values at 2 wt‰, 4 wt‰, and 6 wt‰, but at 8 wt‰ the THR values decreased from 126 to 117 MJ/m^2^, a reduction of 7.1%. In addition, as can be seen from the data in Table 3, the maximum heat radiation rate (MAHRE) decreases as the amount of the flame retardant ECE is added, and the MAHRE value of the PLA flame retardant composite decreases from 295 kW/m^2^ to 279 Kw/m^2^ when the amount of ECE is added at 8 wt‰, which indicates that the PLA/P-PPD-Ph/ECE conjugated flame retardant composite has a high safety in fire (Qian et al., 2015). From the perspective of smoke emission, the total smoke emission (TSR) of PLA flame retardant composites increased from 513 to 827 m^2^/m^2^ as the ECE content increased. In addition, Figure 8C shows that the proportion of CO in the TSR is increasing, indicating that more and more CO is generated, which indicates that the addition of ECE to PLA flame retardant composites can make the matrix material burn inadequately, thus generating more CO and effectively inhibiting the combustion of PLA flame retardant composites. This is because the addition of ECE increases the reactive groups in the PLA flame retardant composite system, which increases the reaction sites between the flame retardant P-PPD-Ph and PLA, thus making the PLA molecular chain with more flame retardant groups and exerting a better flame retardant effect.

**TABLE 3:** Cone calorimeter test data for PLA/P-PPD-Ph/ECE conjugated flame retardant composites.

Samples	TTI	PHRR (kW/m^2^)	MAHRE (kW/m^2^)	THR (MJ/m^2^)	Av-HRR (KJ/m^2^)	Av-EHC (MJ/kg)	TSR (m^2^/m^2^)
PLA/P-PPD-Ph/ECE-0‰	38	452	295	126	221	15	513
PLA/P-PPD-Ph/ECE-2‰	39	443	293	124	219	15	642
PLA/P-PPD-Ph/ECE-4‰	38	466	295	120	222	15	803.8
PLA/P-PPD-Ph/ECE-6‰	39	441	286	121	206	15	812
PLA/P-PPD-Ph/ECE-8‰	40	419	279	117	194	14	827

### 3.6 Char layer analysis of PLA/P-PPD-Ph/ECE conjugated flame retardant composites

**FIGURE 9:**
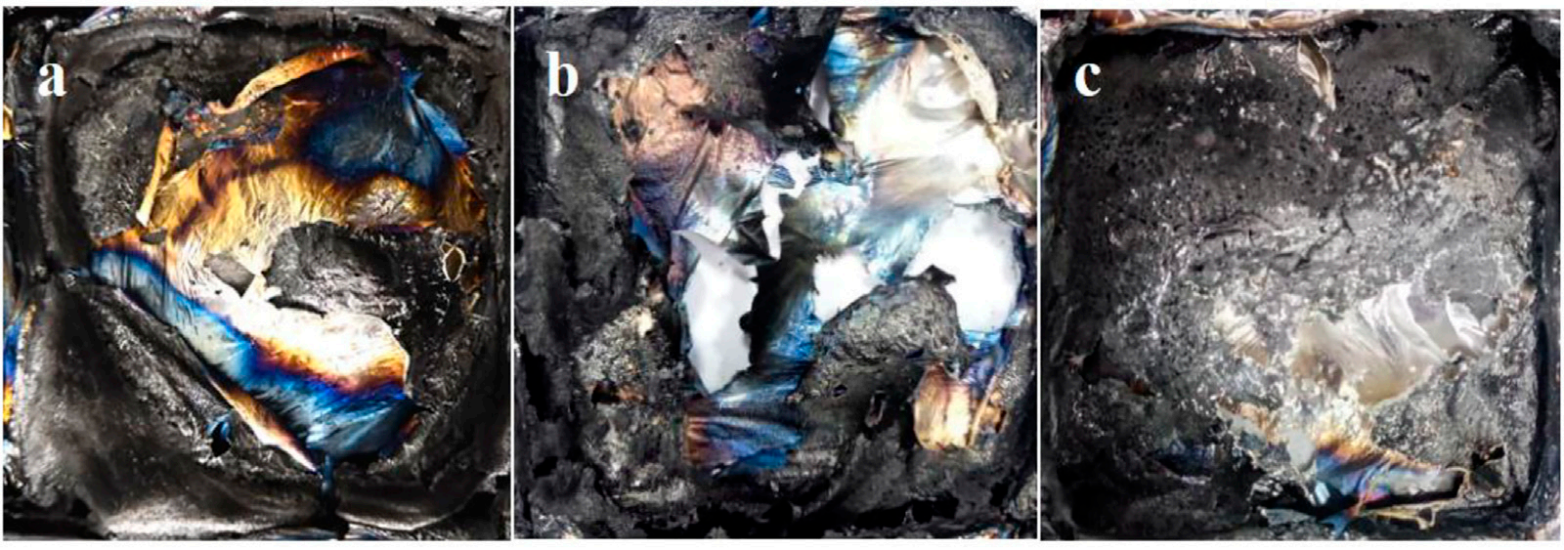
Photograph of the residual carbon layer of the PLA/P-PPD-Ph/ECE flame retardant composite after conical calorimetry: **(A)** PLA/P-PPD-Ph/ECE-0‰, **(B)** PLA/P-PPD-Ph/ECE-4‰, **(C)** PLA/P-PPD-Ph/ECE-8‰.

**FIGURE 10:**
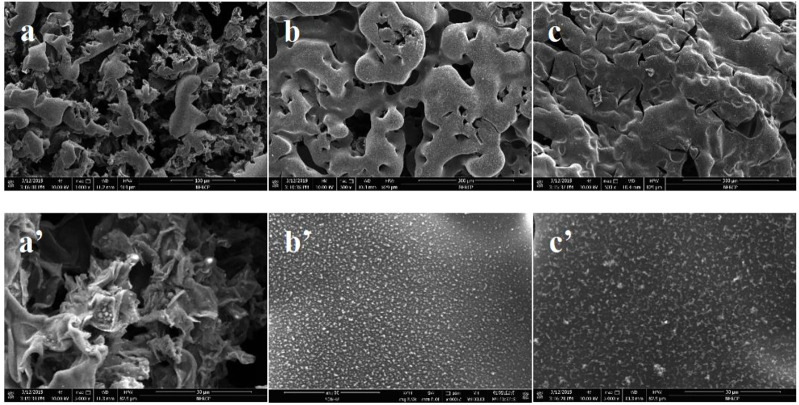
SEM image of residual carbon layer of PLA/P-PPD-Ph/ECE flame retardant composite after cone calorimeter test, **(A)** PLA/P-PPD-Ph/ECE-0‰, **(B)** PLA/P-PPD-Ph/ECE-4‰, **(C)** PLA/P-PPD-Ph/ECE-8‰ (a’) PLA/P-PPD-Ph/ECE-0‰, (b’) PLA/P-PPD-Ph/ECE-4‰, (c’) PLA/P-PPD-Ph/ECE-8‰ are magnified 5000 times.

After conical calorimetric testing, the char layers generated after combustion of PLA flame retardant composites with ECE additions of 0 wt‰, 4 wt‰, and 8 wt‰ were observed. It can be seen from Figure 9 that with a certain amount of flame retardant P-PPD-Ph, more and more char layers were formed after combustion as the amount of ECE added increased. Figure 10 shows the SEM image of the microstructure of the residual char. From the microstructure of the char layer, the char layer formed after combustion of PLA flame retardant composites became more and more dense as the amount of ECE added increased. When the amount of ECE added was 0 wt‰, the char layer was found to be very incomplete, and as the amount of ECE added increased to 4 wt‰, the amount of residual char increased slightly, and when the amount of ECE added reached 8 wt‰, the amount of residual char formed in the PLA flame retardant composite after combustion increased further, and the pores on top of the char layer decreased and the area decreased. The dense char layer can wrap flammable volatiles, thus preventing the release of flammable substances and playing a certain role in flame retardancy. This is because the increase in the amount of ECE added increases the number of reactive functional groups in the PLA flame retardant composites, producing more phosphorus-containing flame retardant groups, increasing the reaction sites between PLA and P-PPD-Ph, resulting in longer molecular chains and higher molecular weight of PLA, cross-linking between PLA and P-PPD-Ph, increasing the denseness of the carbon layer, which to a certain extent improves the flame retardant properties of PLA flame retardant composites (Yang et al., 2015; Xi et al., 2016).

The results of EDS analysis of the char layer of PLA/P-PPD-Ph/ECE conjugated flame retardant composites after combustion are shown in Table 4, which shows that the residual char formed in PLA flame retardant composites after combustion mainly consists of carbon and a small amount of phosphoric acid, with the increase of ECE addition, the C content increases while the O and P content decreases, indicating that the phosphoric acid content in the residue of PLA flame retardant composites after combustion is relatively This indicates that the phosphoric acid content in the residue of the PLA flame retardant composite after combustion is relatively reduced while the carbon residue is relatively increased. This is because with the addition of ECE, a cross-linking reaction occurs in the PLA composite, which strengthens the cohesive phase flame retardant effect of the PLA flame retardant composite, which is corroborated by the analysis of the microstructure of the carbon layer.

**TABLE 4:** Contents of C, O, and P in the EDS test of PLA/P-PPD-Ph/ECE flame retardant composites.

Samples	C (%)	O (%)	P (%)
PLA/P-PPD-Ph/ECE-0‰	73.88	22.85	3.27
PLA/P-PPD-Ph/ECE-4‰	74.85	22.23	2.92
PLA/P-PPD-Ph/ECE-8‰	83.03	15.72	1.25

### 3.7 Mechanical properties testing of PLA/P-PPD-Ph/ECE conjugated flame retardant composites

**FIGURE 11:**
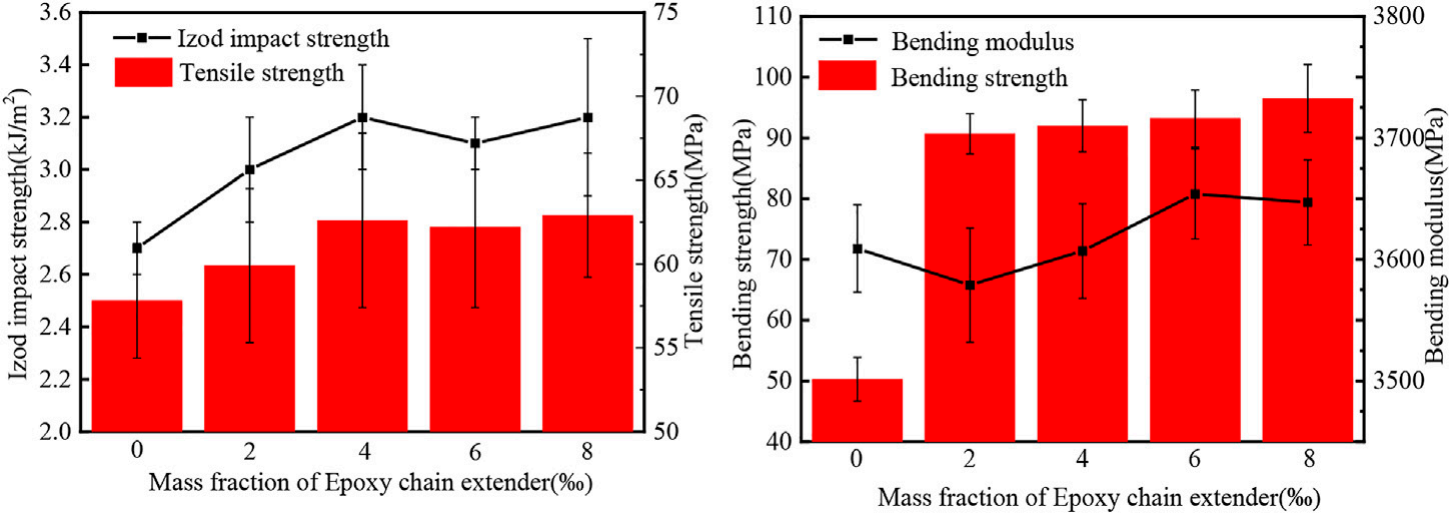
Mechanical properties mapping of PLA/P-PPD-Ph/ECE flame retardant composites.

The bending strength, tensile strength and notched impact strength of the PLA/P-PPD-Ph/ECE conjugated flame retardant composites were characterized by mechanical property tests. Figure 11 and Table 5 show the error bar curve analysis and specific values for the corresponding mechanical property changes. It can be seen that the bending strength, tensile strength and notched impact strength of PLA/P-PPD-Ph/ECE conjugated flame retardant composites all increased with the addition of ECE, and the addition of ECE had a greater effect on the bending strength of PLA flame retardant composites, while it did not have a significant effect on the tensile strength. The bending strength and tensile strength of PLA flame retardant composites were 50.3 and 57.8 MPa, respectively, at 0 wt‰ of ECE addition, and increased to 90.7 and 59.9 MPa, respectively, at 2 wt‰ of ECE addition, an increase of 80% and 3.6%. When the addition of ECE continued to increase, the flexural and tensile strengths of PLA flame retardant composites still showed a small increase. In addition, the overall notched impact strength of PLA flame retardant composites tended to increase with the addition of ECE, but the effect was not significant. When the addition of ECE was 4 wt‰, the notched impact strength increased from the initial 2.7 kJ/m^2^ to 3.2 kJ/m^2^, an increase of 1.9%. In summary, the addition of ECE led to an increase in the mechanical properties of the PLA flame retardant composites.

**TABLE 5:** Mechanical properties of PLA/P-PPD-Ph/ECE flame retardant composites.

Sample	Bending strength (MPa)	Bending modulus (MPa)	Tensile strength (MPa)	Izod impact strength (kJ/m^2^)
PLA/P-PPD-Ph/ECE-0‰	50.3 ± 3.6	3609 ± 36	57.8 ± 3.4	2.7 ± 0.1
PLA/P-PPD-Ph/ECE-2‰	90.7 ± 3.3	3579 ± 47	59.9 ± 4.6	3.0 ± 0.2
PLA/P-PPD-Ph/ECE-4‰	92.0 ± 4.3	3607 ± 39	62.6 ± 5.2	3.2 ± 0.2
PLA/P-PPD-Ph/ECE-6‰	93.2 ± 4.7	3654 ± 37	62.2 ± 4.8	3.1 ± 0.1
PLA/P-PPD-Ph/ECE-8‰	96.5 ± 5.6	3647 ± 35	62.9 ± 3.7	3.2 ± 0.3

## Conclusion

In this paper, PLA/P-PPD-Ph/ECE conjugated flame retardant composites were prepared using ECE as a chain extender for PLA and P-PPD-Ph successfully prepared in the laboratory as a flame retardant for PLA, and the effects of different additions of ECE on the thermal properties, flame retardant properties and mechanical properties of PLA/P-PPD-Ph/ECE conjugated flame retardant composites were investigated. The results showed that the addition of ECE increased the maximum thermal decomposition temperature of the composites from 394.0°C to 395.9°C, the residual carbon rate around 700°C increased from 1.6% to 3.3%, and the thermal stability was improved. From the structural formula of ECE, it can be seen that ECE in the composites cross-linked lightly with PLA and P-PPD-Ph and formed a mesh structure, which increased the cross-linking of PLA and made the PLA molecular chain with more flame retardant groups, SEM and EDS analysis showed that the carbon layer of the composites became more dense, C increased while P decreased, and when the addition amount of ECE was 8 wt‰, The LOI of the PLA/P-PPD-Ph/ECE flame retardant composites increased from 29.8% to 32.6%, an increase of 9.4%. The values of PHRR, THR and Av-EHC decreased, and the amount of TSR and generated CO increased significantly, indicating that ECE played a cohesive phase flame retardant role in the composites. In addition, during the melt processing, the epoxy groups contained in the ECE molecular chain can react with the hydroxyl and carboxyl groups at the end of the PLA molecular chain, increasing the molecular weight of PLA and improving the mechanical properties of the composites. The results of this study lay the foundation for increasing the flame retardancy of the material by adding epoxy chain extenders to increase the crosslinking between the flame retardant and the matrix in the future, in addition to increasing the processing and application of PLA biodegradable materials in fire resistant products such as electronic appliances, automobiles, construction and textiles.

## Data Availability

The original contributions presented in the study are included in the article/Supplementary Material, further inquiries can be directed to the corresponding authors.
